# The K-Ras(G12D)-inhibitory peptide KS-58 suppresses growth of murine CT26 colorectal cancer cell-derived tumors

**DOI:** 10.1038/s41598-022-12401-3

**Published:** 2022-05-17

**Authors:** Kotaro Sakamoto, Bangzhong Lin, Kazuto Nunomura, Takeshi Izawa, Shinsaku Nakagawa

**Affiliations:** 1 Research & Development Department, Ichimaru Pharcos Company Limited, 318-1 Asagi, Motosu, Gifu 501-0475 Japan; 2 Center for Supporting Drug Discovery and Life Science Research, Graduate School of Pharmaceutical Science, Osaka University, 1-6 Yamadaoka, Suita, Osaka 565-0871 Japan; 3 Laboratory of Veterinary Pathology, Osaka Prefecture University, 1-58 Rinku-Orai-Kita, Izumisano, Osaka 598-8531 Japan; 4 Laboratory of Biopharmaceutics, Osaka University, 1-6 Yamadaoka, Suita, Osaka 565-0871 Japan

**Keywords:** Cancer therapy, Gastrointestinal cancer, Oncogenes, Cancer, Drug discovery, Drug safety, Pharmacology, Target validation, Toxicology

## Abstract

Mutations in the cell proliferation regulator K-Ras are found with a variety of cancer types, so drugs targeting these mutant proteins could hold great clinical potential. Very recently, a drug targeting the K-Ras(G12C) mutant observed in lung cancer gained regulatory approval and several clinical trials are currently underway to examine the efficacy of this agent when combined with other drugs such as a monoclonal antibody inhibitor of programmed cell death 1 receptor (anti-PD-1). Alternatively, there are currently no approved drugs targeting K-Ras(G12D), the most common cancer-associated K-Ras mutant. In 2020, we described the development of the K-Ras(G12D) inhibitory bicyclic peptide KS-58 and presented evidence for anticancer activity against mouse xenografts derived from the human pancreatic cancer cell line PANC-1 stably expressing K-Ras(G12D). Here, we show that KS-58 also possess anticancer activity against mouse tumors derived from the colorectal cancer cell line CT26 stably expressing K-Ras(G12D). Further, KS-58 treatment reduced phosphorylation of ERK, a major downstream signaling factor in the Ras pathway, confirming that KS-58 inhibits K-Ras(G12D) function. Unexpectedly; however, KS-58 did not show additive or synergistic anticancer activity with mouse anti-PD-1. Morphological analysis and immunostaining demonstrated no obvious differences in CD8^+^ cells infiltration or PD-L1 expression levels in CT26-derived tumors exposed to monotherapy or combination treatment. Nonetheless, KS-58 demonstrated reasonable stability in blood (*t*_1/2_ ≈ 30 min) and no obvious systemic adverse effects, suggesting clinical potential as a lead molecule against colorectal cancer.

## Introduction

The Ras/MAPK signaling pathway protein K-Ras is a highly conserved molecular switch regulating cell proliferation, and several mutations in the K-Ras amino acid sequence, including K-Ras(G12D), K-Ras(G12V), and K-Ras(G12C), and have detected in various types of human cancer^1^. Therefore, molecular inhibitors targeting these mutants may be broadly effective antitumor agents. Recently, an irreversible covalent inhibitor against K-Ras(G12C), a mutation detected mainly in lung cancer, was granted regulatory approval based on clinical trial data showing efficacy as monotherapy^2,3^. In addition, several ongoing clinical trials are examining the efficacy of K-Ras(G12C) inhibitor in combination with other drugs, including immune checkpoint inhibitors (such as a monoclonal antibody against programmed cell death 1 receptor or anti-PD-1) and various Ras pathway inhibitors (e.g., EGFR, MEK, SHP2, and SOS1 inhibitors)^4,5^, as K-Ras(G12C) inhibitor plus anti-PD-1 exerted synergistic anticancer effects in preclinical studies^6^.

**Figure 1:**
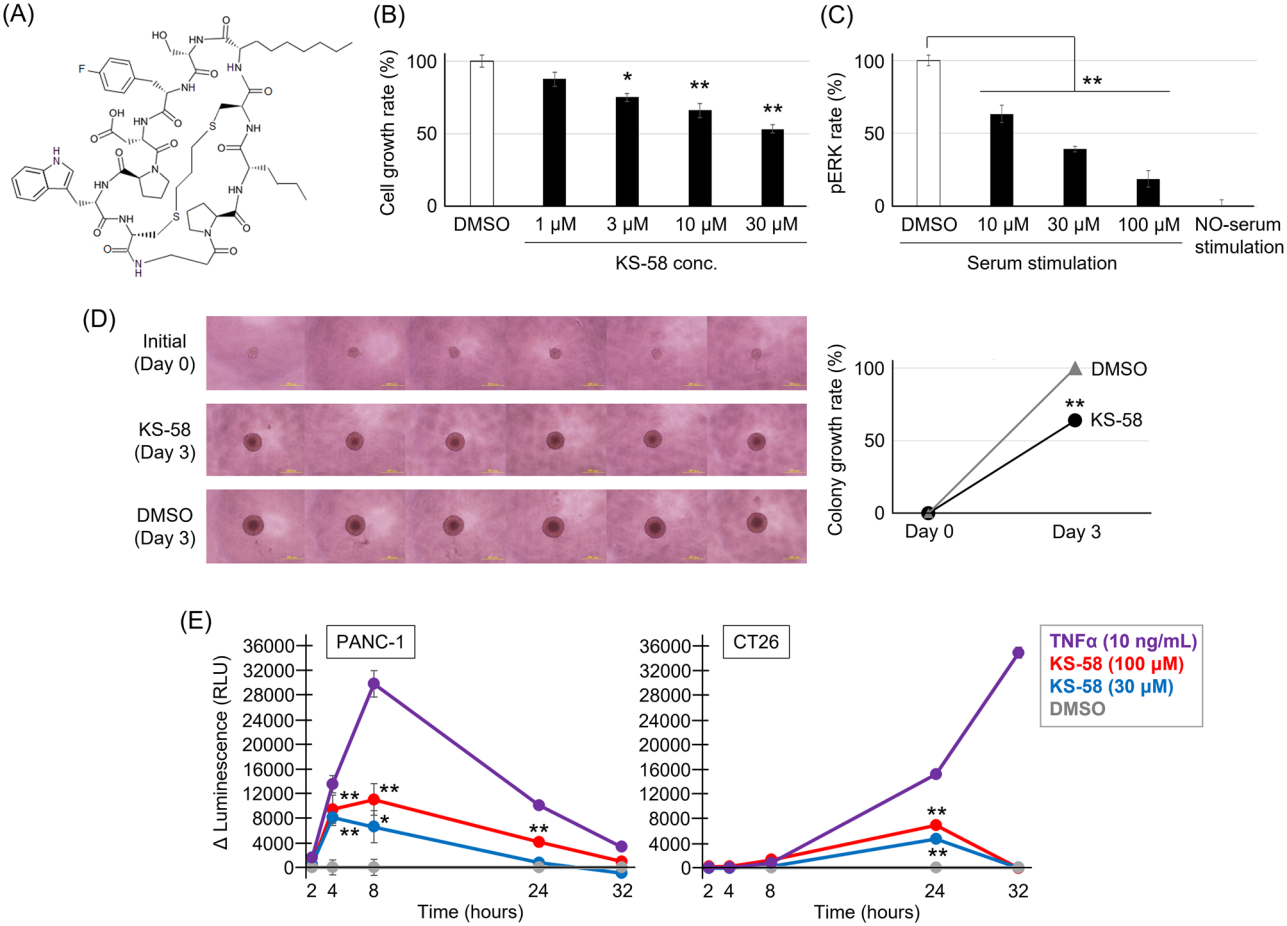
KS-58 suppressed in vitro proliferation of the murine colorectal cancer cell line CT26. (**A**) Chemical structure of KS-58. (**B**) Dose-dependent suppression of CT26 cell proliferation by KS-58. Results in (**B**) are expressed as mean ± S.E.M of n = 4 independent experiments using separately treated cultures (*p < 0.05 and **p < 0.01 vs. DMSO by Dunnett’s test). (**C**) Dose-dependent suppression of phosphorylated ERK (pERK) in CT26 cells by KS-58 (n = 6, mean ± S.E.M, **p < 0.01 vs. DMSO control by Dunnett’s test). The phosphorylation levels were shown as % value compared to DMSO set as 100% and no-serum stimulation set as 0%. (**D**) Colony growth suppression activity of KS-58 (30 μM) (yellow bar: 50 μm, n = 6, mean ± S.E.M, **p < 0.01 vs. DMSO control by Student’s t-test). The colony growth levels were shown as % value compared to DMSO at day 3 set as 100% and initial at day 0 set as 0%. (**E**) Apoptosis inducing effect of KS-58 (30 μM and 100 μM) (n = 8, mean ± S.E.M, *p < 0.05 and **p < 0.01 vs. DMSO by Dunnett’s test). The value on vertical axis means actual values minus average value of DMSO control.

However, the therapeutic spectrum of K-Ras(G12C) inhibitor is limited because this mutation is found in only a minority of non-small cell lung cancers and rarely in other cancer types. In contrast, K-Ras(G12D) and K-Ras(G12V) are frequently detected in intractable tumors of the pancreas, colon, and rectum^7,8^, but attempts to produce proteins that inhibit these mutants via irreversible covalent binding have been unsuccessful^9,10^. In 2017, the first K-Ras(G12D)-selective inhibitory peptide KRpep-2d was reported^11–13^, and subsequently demonstrated to reversibly bind K-Ras(G12D) and suppress in vitro proliferation of human A427 lung carcinoma cells expressing K-Ras(G12D). In 2020, an optimized KRpep-2d was described, the bicyclic peptide KS-58 (Fig. 1A)^14^, and shown to suppress the growth of xenograft mouse tumors derived from the human pancreas carcinoma cell line PANC-1. It was also found that combination treatment of KS-58 plus gemcitabine exerted additive anticancer activity in vivo. Further, molecular dynamics simulations suggested that conformational changes allow KS-58 to access and move within the lipid bilayer membrane despite its size (1333 g/mol) (data not shown).

In the current study, we examined the anticancer activity of KS-58 against mouse colorectal tumors derived from the murine cell line CT26 stably expressing K-Ras(G12D), both when administered alone and with the immune checkpoint inhibitor mouse PD-1 antibody (anti-PD-1). We also examined the pharmacokinetics of KS-58 in mice and K-Ras(G12D) targeting in tumors by measuring changes in the phosphorylation of ERK, a downstream signal in the Ras-Raf-MEK pathway.

## Materials and methods

### In vitro cell proliferation assay, ERK-phosphorylation assay, colony growth assay, and apoptosis assay

KS-58 was produced by SCRUM Inc. (Tokyo, Japan) using Fmoc-based solid phase peptide synthesis as previously described^14^. The CT26.WT (CRL-2638) mouse colorectal cancer cell line was purchased from American Type Cell Collection (ATCC) (Manassas, VA, USA), and maintained in RPMI-1640 supplemented with 10% fetal bovine serum (growth medium) at 37 °C under a humidified 5% CO_2_ atmosphere according to the manufacturer’s recommended protocol.

Cell proliferation was measured using the CellTiter-Glo assay kit (G7570, Promega, WI, USA) according to the manufacturer’s instructions as previously described^14^. Briefly, cells were seeded at 1,000/well in 96-well tissue culture plates with growth medium and allowed to adhere overnight (day 0). For cell treatment, KS-58 was first solved in DMSO and then diluted in growth medium to a constant final DMSO concentration of 0.5%. Cells were incubated in the indicated treatment medium for 3 days with daily medium replacement. Relative cell numbers were estimated using a SpectraMax i3x luminescence detector (Molecular Devices, San Jose, CA, USA). Cell proliferation rate (%) was calculated relative to baseline on day 0 (0%) and day 3 without peptide treatment (100%).

For evaluation of suppressing effects of KS-58 on Ras-ERK pathway in CT26 cells, phosphorylated ERK (pERK) was measured by Phospho-ERK1 (T202/Y204)/ERK2 (T185/Y187) DuoSet IC ELISA (DYC1018B-2, R&D Systems) according to the protocol supplied by the manufacture. CT26 cells were starved for 16 h, treated with KS-58 which was first solved in DMSO and then diluted in FBS-free medium for 1 h (a constant final DMSO concentration of 1.0%), and then treated with peptide diluted in FBS-containing medium for 1 h (a constant final FBS concentration is 10%). Phosphorylation rate of ERK (%) was calculated based on no serum stimulation as 0% and serum stimulation without peptide as 100%.

For colony growth assay, CT26 cells were seeded at 500 cells/well in 96-well plates (7007, COSTER) for 3D-culture. After 24 h incubation, the initial size (day 0) of colonies was measured as area using ImageJ (NIH, Bethesda, MD, USA). For colony treatment, KS-58 was first solved in DMSO and then diluted in growth medium to a constant final DMSO concentration of 0.5%. Colonies were incubated in the indicated treatment medium for 3 days with daily medium replacement. The size changes of colonies were measured at day 3 and the size changes rate (%) was calculated relative to initial on day 0 (0%) and day 3 without peptide treatment (100%).

For apoptosis assay, RealTime-Glo Annexin V Apoptosis Assay reagent (JA1000, Promega) was used according to the protocol supplied by the manufacture. PANC-1 (ATCC CRL-1469) and CT26 cells were seeded at 10,000 cells/well in 96-well tissue culture white plates with 10% FBS culture mediums. After 16 h incubation, KS-58 and apoptosis assay reagents diluted in FBS-containing medium were added to the wells. Luminescence (apoptosis signal) were measured at 0, 2, 4, 8, 24 and 32 h after KS-58/reagents addition. THFα (10 ng/mL) (HZ-1014, Humanzyme) was used as a positive control of apoptosis inducer.

### Measurement of in vivo anticancer activity

Experimental procedures that involved animals and their care were conducted in compliance with the *Guide for the Care and Use of Laboratory Animals*^15^ and ARRIVE guidelines^16,17^. Animal experiments were approved by Animal Care and Use Committee of UNITECH Co. Ltd. (Kashiwa, Japan) (approval No. AGR IMF-210415A-30) and conducted at UNITECH in accordance with the guidelines. To establish subcutaneous allograft tumors, 1 × 10^6^ CT26 cells/100 µL were subcutaneously injected into the left groin region of BALB/cCrSlc female mice (7 weeks old; Japan SLC, Inc., Shizuoka, Japan). Immediately before treatment, KS-58 was dissolved in DMSO and then diluted tenfold in normal saline. Tumor-baring mice in vehicle control, KS-58 (low), and KS-58 (high) groups were injected intravenously with vehicle (1:10 DMSO to saline, v/v), 10 mg/kg KS-58 solution, or 40 mg/kg KS-58 solution, respectively, once every 2 days for 17 days (n = 8 mice per major group, n = 3 mice per satellite group) (Fig. 1B). For anti-PD-1 monotherapy or combination treatment, GoInVivo Purified anti-mouse PD-1 (CD279, Cat.#114114, BioLegend, San Diego, CA, USA) was dissolved in PBS and injected intraperitoneally at 50 mg/kg on days 6, 8, and 10 (n = 8 mice in major groups, n = 3 in satellite groups). Tumor growth rate was recorded on day 1, 4, 8, 11, 15, and 17 by measuring the major and minor axes with a digital caliper. Measurements were transformed into tumor volumes using the formula tumor volume (mm^3^) = major axis × minor axis^2^ × 0.5. Tumors (with surrounding skin), liver, and kidneys were collected from satellite groups on day 15, fixed in formalin, and then maintained in 70% ethanol for histological analyses. Tumor, liver, and kidneys were collected from major groups on day 17 within 3 h of the last injection and weighed. The tumors were subsequently frozen in liquid nitrogen and stored at − 80 °C until homogenization for Western blotting.

### Western blotting

Experiments were conducted at UNITECH. Briefly, frozen tumors excised as described were homogenized in cold RIPA buffer (182-02451, FUJIFILM Wako Pure Chemical Co., Osaka, Japan) supplemented with cOmplete mini EDTA-free Protease Inhibitor Cocktail (11836170001, Roche, Basel, Switzerland) and PhosSTOP using a BioMasher II disposable homogenizer (Nippi, Inc., Tokyo, Japan), incubated on ice for 30 min, and then centrifuged. The supernatant containing target proteins was collected and total protein concentration was measured. Supernatant proteins (30 μg per gel lane) were separated by SDS-PAGE using Laemmli mini gels (90 mm × 80 mm) and electrophoretically transferred onto polyvinylidene difluoride membranes. Membranes were blocked in TBS containing 0.1% Tween 20 (TBST) plus 3% BSA for 1 h at room temperature (RT) and then incubated with a rabbit monoclonal antibody against phospho-p44/42 MAPK (Erk1/2) (Thr202/Tyr204) (1000-fold dilution) (20G11, Cell Signaling Technology, Danvers, MA, USA) in TBST plus 1% BSA for 1 h at RT. Blotted membranes were then incubated with horseradish peroxidase (HRP)-conjugated mouse anti-rabbit IgG (4000-fold dilution) (4090-05, SouthernBiotech, Birmingham, AL, USA) in TBST plus 1% BSA for 1 h at RT. The reactions were detected with ImmunoStar Zeta (FUJIFILM Wako) and Hyperfilm ECL (Cytiva, Marlborough, MA, USA). After detection, membranes was incubated with stripping buffer (62.5 mM Tris–HCl, 2% SDS, 100 mM 2-mercaptoethanol, pH 6.5) for 30 min at 50 °C and then washed five times with TBST for measurement of total ERK as described above using p44/42 MAPK (Erk1/2) rabbit monoclonal antibody (1000-fold dilution) (137F5, Cell Signaling Technology). After detection, membranes was incubated with stripping buffer (62.5 mM Tris–HCl, 2% SDS, 100 mM 2-mercaptoethanol, pH 6.5) for 30 min at 50 °C and then washed five times with TBST for measurement of GAPDH as described above using anti-GAPDH antibody (1000-fold dilution) (ab8245, abcam). Protein bands were quantified using ImageJ (NIH, Bethesda, MD, USA). Full length blots are shown in Supplementary Fig. S1.

### Pharmacokinetics, stability in liver microsomes, stability in whole blood, translocation to blood cells, and plasma protein binding evaluations

Experimental procedures that involved animals and their care were conducted in compliance with the *Guide for the Care and Use of Laboratory Animals*^15^ and ARRIVE guidelines^16,17^. Animal experiments were approved by the Animal Care and Use Committee of Osaka University (approval No. DouyakuR01-3–1) and conducted at Osaka University in accordance with the guidelines. LC–MS/MS data were obtained using a Xevo TQ-S mass spectroscopy system (Waters Corp., Milford, MA, USA) connected to an ACQUITY UPLC system (Waters) equipped with a BEH C18 column (1.7 μm, 2.1 × 50 mm, Waters). Mobile phase A was 0.1% formic acid in water and mobile phase B was 0.1% formic acid in acetonitrile. The gradient elution profile was 2% B at 0 min to 98% B at 1.8 min, and the flow rate was 0.5 mL/min.

For pharmacokinetics studies, KS-58 was dissolved in saline and administered intravenously to female BALB/c mice at 10 mg/kg. Serial blood samples (20 μL) were collected from the tail vein using heparinized tips at 7.5, 15, 30, 60, 120, 240, 320, and 1440 min after KS-58 administration. Aliquots (5 μL) of plasma obtained from each blood sample were treated with 50 μL acetonitrile and the organic layer was injected onto the LC–MS/MS system. Pharmacokinetic parameters were obtained by fitting the plasma concentration–time data to a noncompartmental model using PKPlus software (Northern Science Consulting Inc, Sapporo, Japan).

For evaluation of stability, KS-58 was incubated with commercial hepatic microsomes (XenoTech Ltd, KS, USA) in X medium supplemented with nicotinamide adenine dinucleotide phosphate for 10 and 60 min at 37 °C. The fraction (%) remaining in the medium was determined using the LC–MS/MS system (Waters). We also examined the fraction (%) remaining in fresh mouse blood after 60 and 120 min of incubation at 37 °C using the same LC–MS/MS system.

For evaluation of plasma protein binding, the plasma compartment containing KS-58 was dialyzed against PBS (pH 7.4) for 5 h at 37 °C and the concentrations remaining in each compartment were quantified by LC–MS/MS.

### Immunostaining and morphological analysis of tumors, livers, and kidneys

Tumors, livers, and kidneys were harvested from mice and fixed with 10% neutral-buffered formalin. After 24 h, tissues were transferred to 70% ethanol and stored at RT until analyses. Tissues were subsequently paraffinized, sectioned at 5-μm thickness, deparaffinized in xylene, dipped in 100% ethanol, washed in water, and then stained with antibodies or HE.

For immunostaining, the sections were autoclaved for 10 min at 121 °C in 10 mM Tris/1 mM EDTA (pH 9.0) for antigen retrieval, blocked with 5% skim milk in PBS for 60 min at RT, and incubated with specific primary antibodies against the effector T-cell marker CD8 (1:2,000 dilution, #ab217344, Abcam, Cambridge, UK) and PD-L1 (E1L3N) XP (1:200 dilution, #13684, Cell Signaling Technology) for 60 min. Subsequently, the sections were treated with 3% H_2_O_2_ in PBS to quench endogenous peroxidase activity and incubated with Simple Stain MAX PO (1:3 dilution, Nichirei Bioscience Inc., Tokyo, Japan) for 60 min at RT. Sections were then stained with DAB solution (Nichirei Bioscience Inc.), washed thoroughly with PBS, counterstained with hematoxylin for 1 min, washed with water, dehydrated, mounted, and scanned using a VS-120 Slide Scanner (Olympus, Tokyo, Japan).

For morphological analysis, sections cut at 5 μm were stained with hematoxylin and eosin. Histopathological analysis was conducted by a board-certified veterinary and toxicologic pathologist (T.I., Diplomate of the Japanese College of Veterinary Pathology and Japanese Society of Toxicologic Pathology).

### Data analysis

Software used for the statistical analysis was JMP8 (SAS Institute Inc.) The level of significance was set at 5% (*p* < 0.05).

## Results

### KS-58 suppressed the proliferation of mouse CT26 colorectal cancer cells in vitro

It was reported that KS-58 (Fig. 1A) suppresses growth of the human lung cancer cell line A429 and human pancreatic cancer cell line PANC-1 stably expressing K-Ras(G12D)^14^. On the other hand, effects on the proliferation of colorectal cancer cells, which frequently express K-Ras(G12D)^18^, have not been reported. Here we demonstrate that KS-58 dose-dependently suppresses the proliferation of murine CT26 colorectal cancer cells stably expressing K-Ras(G12D), with about 50% inhibition at 30 μM (Fig. 1B). KS-58 significantly down-regulated phosphorylation of ERK in CT26 cells (Fig. 1C) and reduced growth of CT26 cell colonies (Fig. 1D). These data suggests that KS-58 inhibits K-Ras downstream signaling. However, its growth suppression activity requires micromolar order, which deviates from nanomolar order K-Ras-binding activity^14^. This may be due to the low cell membrane permeability of KS-58. Although the number of cells at the endo of test was not lower than at the beginning of test (both Fig. 1B,D), it was of interest whether K-Ras(G12D) inhibition by KS-58 induces apoptosis or not. To confirm this, apoptosis assay against PANC-1 and CT26 cells was done. As a result, KS-58 induced apoptosis in PANC-1 and CT26 cells in a concentration-dependent manner within 24 h. However, the degree of apoptosis induction by KS-58 was mild compared to that of TNFα (10 ng/mL) known as an apoptosis inducer.

### Anticancer effects of KS-58 on mice CT26 allografts

**Figure 2:**
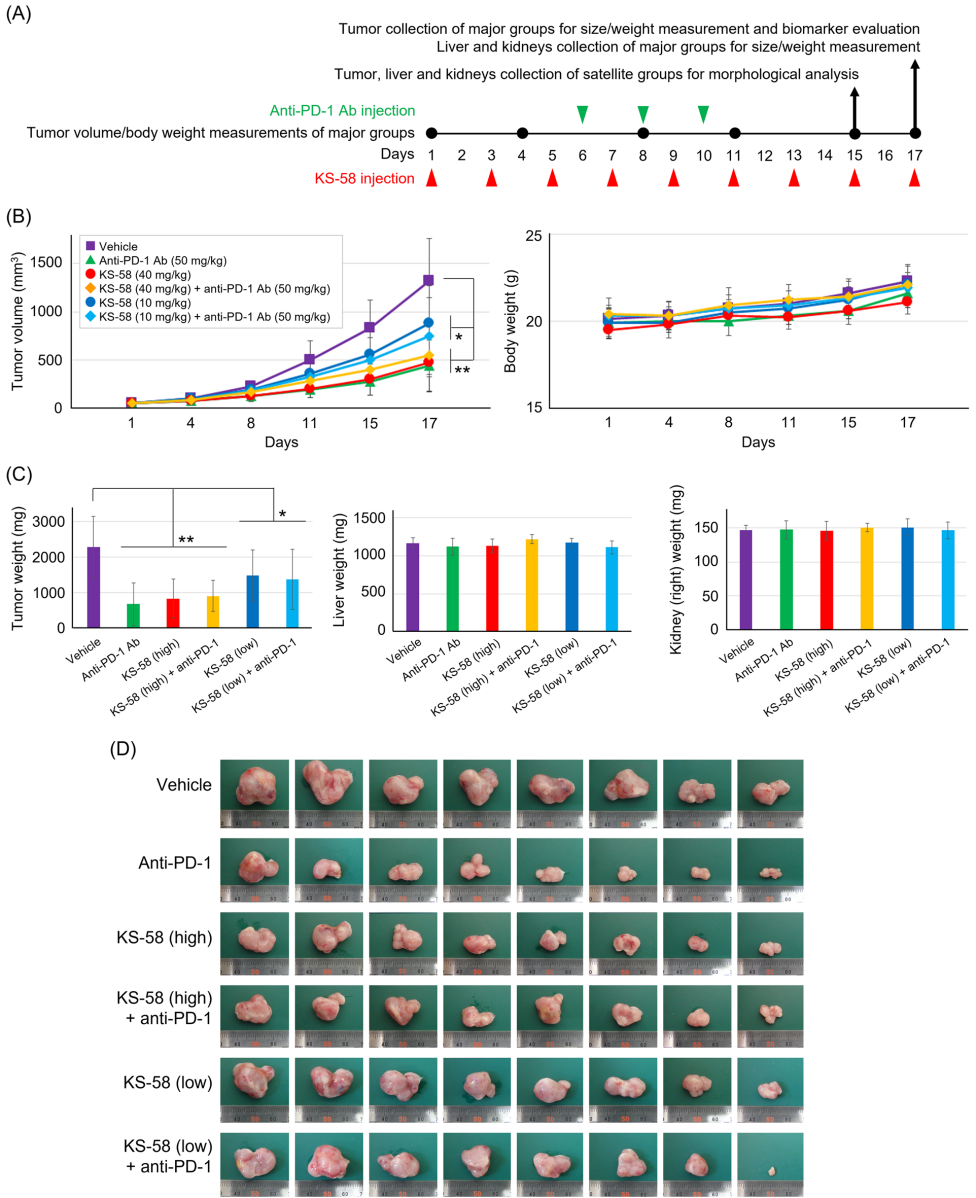
Anticancer activities of KS-58 alone and KS-58 plus anti-PD-1 antibody against CT26 allografts. (**A**) Schematic of experimental procedures. Major groups (n = 8 mice per group) were tested to evaluate anti-cancer activity. Tumors, livers and kidneys of satellite groups (n = 3 mice per group) were collected for morphological analysis of Fig. 4. (**B**) Changes in tumor volume and body weight for each major treatment group (n = 8 mice per group, expressed as mean ± SD, *p < 0.05 and **p < 0.01 vs. Vehicle control by Dunnett’s test). (**C**) Weights of tumor, liver, and kidney (right) collected from major groups on day 17 (n = 8 mice per group, mean ± SD, *p < 0.05 and **p < 0.01 vs. Vehicle control by Dunnett’s test). (**D**) Appearance of tumors collected from major groups. KS-58 doses were 40 mg/kg (high) and 10 mg/kg (low) in all experiments.

We next evaluated the anticancer activity of KS-58 in mice subcutaneously injected with CT26 cells stably expressing K-Ras(G12D) (Fig. 2A). Tumor size increased progressively following transplantation, but the rate of growth was substantially reduced by subsequent treatment with KS-58 (10 or 40 mg/kg every second day) or anti-PD-1 (50 mg/kg on days 6, 8, and 10 post-transplant) compared to vehicle treatment (Fig. 2B). Unexpectedly, however, there was little difference in tumor size between mice receiving KS-58 alone and those receiving the combination of KS-58 plus anti-PD-1 (Fig. 2B,D), with tumors exposed to high-dose KS-58 showing a 65% weight reduction compared to controls and tumors exposed to both high-dose KS-58 plus anti-PD-1 showing a 55% reduction (Fig. 2C,D). While KS-58 did not augment the antitumor activity of anti-PD-1, it did reduce tumor weight without inducing systemic adverse effects as neither body weight gain/loss (Fig. 2B) nor the weights of liver and kidney (Fig. 2C) were significantly altered compared to vehicle controls.

### Treatment with KS-58 reduced intratumoral expression of phosphorylated ERK, a biomarker of K-Ras(G12D) inhibition

**Figure 3:**
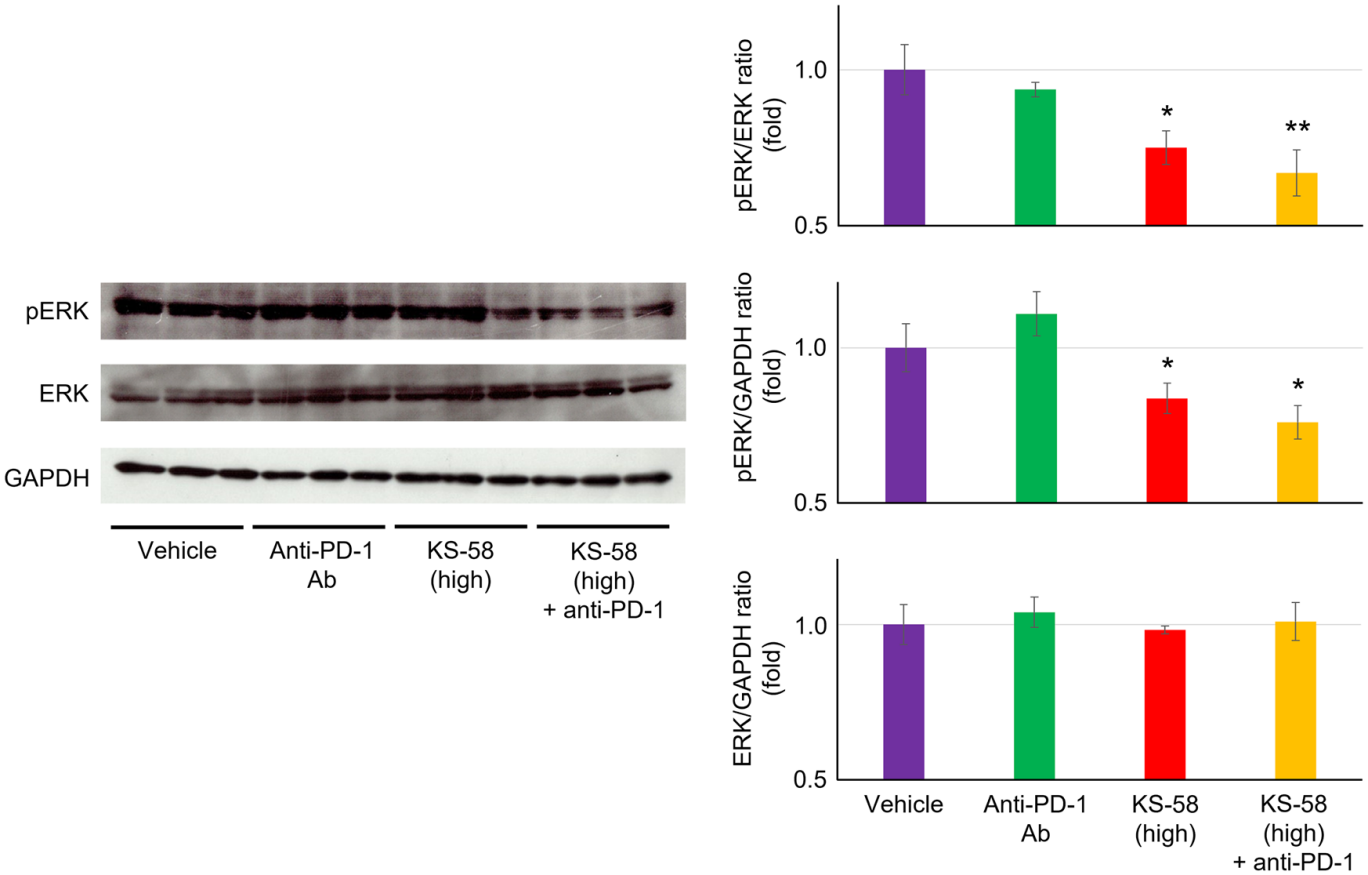
Inhibition of K-Ras(G12D) in CT26 cell-derived tumors by KS-58 measured using phosphorylated ERK as a biomarker. Tumors collected from major groups within 3 h after peptide or vehicle injection were homogenized, and phosphorylated ERK (pERK), total ERK and GAPDH were quantified by Western blotting. Expression levels of pERK were normalized by total ERK and GAPDH, respectively. Expression levels of total ERK was normalized by GAPDH. These expression levels are presented as the fold change relative to the vehicle group (n = 3 tumors per group, mean ± S.E.M, *p < 0.05 and **p < 0.01 vs. Vehicle by Dunnett’s test).

To confirm that the in vivo anticancer activity of KS-58 was caused by K-Ras(G12D) inhibition, we evaluated phosphorylation of ERK (pERK), a downstream components of the K-Ras signaling cascade. Possible methods to evaluate pERK in tumors are (1) homogenizing tumor and evaluating the pERK by ELISA or Western blotting, and (2) preparing tissue sections of tumor and evaluating the pERK by immunohistochemical (IHC) staining. We first attempted to examine by Western blotting. Three tumors were randomly selected from each of the four major treatment groups (vehicle, anti-PD-1 antibody, 40 mg/kg KS-58, and 40 mg/kg KS-58 plus anti-PD-1) (a total of 12 tumors), homogenized, and subjected to Western blotting to quantify pERK, total ERK and GAPDH (Fig. 3). The pERK/ERK ratio and pERK/GAPDH ratio were significantly reduced in both KS-58 mono- and combination groups but not in the anti-PD-1 group. Additionally, ERK/GAPDH ration was not significantly changed in any groups in compared with vehicle treatment. This result suggests that (1) the in vivo anticancer activity of KS-58 demonstrated in Fig. 2 results from K-Ras(G12D) inhibition and (2) anti-PD-1 antibody does not attenuate the in vivo K-Ras(G12D) inhibitory activity of KS-58. Although pERK evaluation by IHC staining was not performed due to the success of pERK evaluation by Western blotting, IHC staining would be necessary in the future to clarify the details of the in vivo anti-tumor effects of KS-58 including ERK signaling, proliferation and apoptosis of tumor cells.

### Pharmacokinetics, stability, blood cell translocation, and plasma protein binding of KS-58

**Figure 4:**
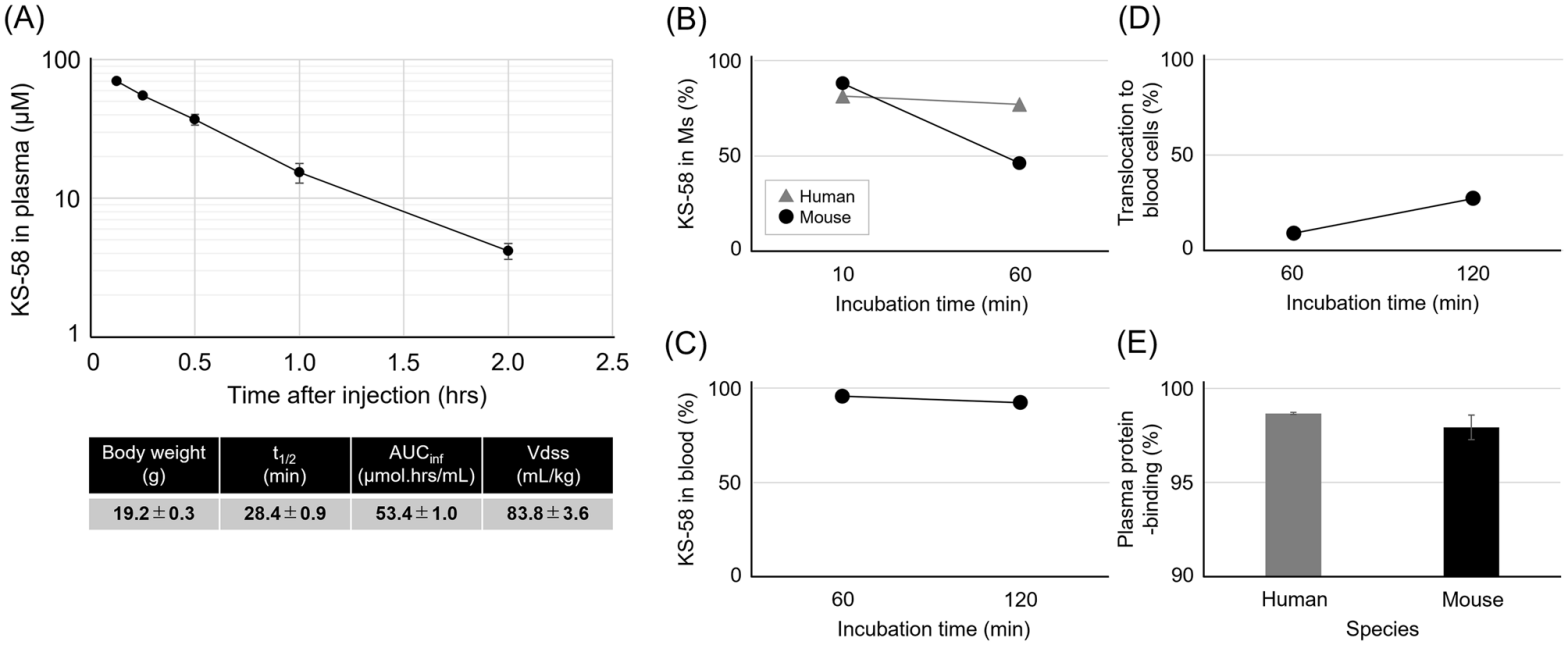
Pharmacokinetics and stability of KS-58. (**A**) KS-58 was intravenously injected at 10 mg/kg (n = 3 mice) and blood samples were collected at the indicated times. The concentration of KS-58 (mean ± SD) in plasma was determined by LC–MS/MS and the pharmacokinetics parameters t_1/2_, AUC_inf_, and Vdss were calculated as described in “Materials and methods” section. (**B**) KS-58 remaining in human and mouse liver microsomes (Ms) following 10 and 60 min of incubation. (**C**) Residual KS-58 in mouse whole blood after 60 and 120 min of incubation. (**D**) Translocation of KS-58 to blood cells. (**E**) Binding (%) of KS-58 to human and mouse plasma proteins.

In our previous study^14^, we demonstrated the serum protease resistance of KS-58 and estimated the half-life in whole blood (~ 60 min) using RP-HPCL. Here, we evaluated the pharmacokinetics of KS-58 in greater detail using LC–MS/MS. The estimated limit of detection was ~ 250 nM. Following injection of 10 mg/kg, the plasma concentration decreased to ~ 50% of the initial concentration after about 30 min and was below the limit of detection after 4 h (Fig. 4A). Detailed pharmacokinetic parameters including t_1/2_, area under the concentration–time curve from time zero to infinity (AUC_inf_), and the volume of distribution at steady-state (Vdss) are shown in Fig. 4A.

The stability of KS-58 was evaluated in human and mouse liver microsomes as well as in whole blood (Fig. 4B,C). After 1 h of incubation, 77% of the initial concentration remained in human liver microsomes but only 46.2% remained in mouse liver microsomes. In whole blood, 92.3% of the initial concentration was intact even after 2 h, consistent with plasma protease resistance. In addition to degradation, the concentration decrease in vivo may reflect compartmentalization in blood cells and (or) protein binding. In fact, a substantial fraction of KS-58 was translocated into blood cells (Fig. 4D). Further, plasma protean binding was high, as less than 10% of KS-58 mixed with plasma was in the free state and more than 90% was bound to plasma proteins after 5 h of incubation.

### Effects of KS-58 on tumor effector CD8^+^ cells infiltration and PD-L1 expression

**Figure 5:**
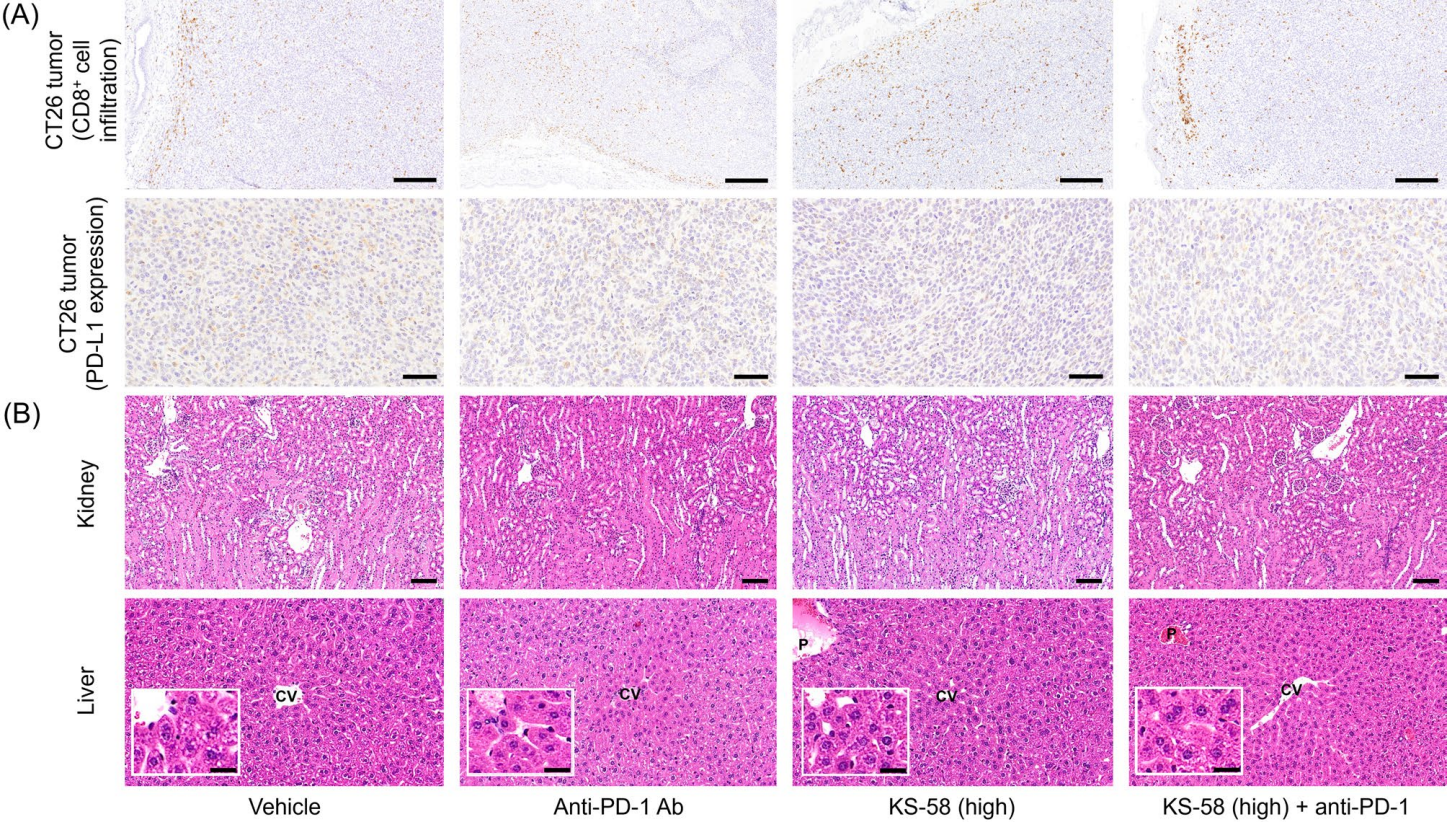
Effects of KS-58 alone and combined with anti-PD-1 on tumor and non-target organ histopathology. Tumors, livers, and kidneys collected from satellite mouse groups were fixed in 10% neutral-buffered formalin, embedded in paraffin, sectioned, and stained with hematoxylin and eosin. (**A**) CD8^+^ cells infiltration (black bar: 200 μm) and PD-L1 expression (black bar: 50 μm) were evaluated by immunostaining. (**B**) Cell degeneration and necrosis were evaluated by morphological analysis using light microscopy (black bar: 100 μm in kidney and 20 μm in liver). *CV* central vein, *P* portal area in liver.

These blood cell translocation measurements (Fig. 4D) suggest that KS-58 can influence the functioning of blood cells including lymphocytes. We speculated that KS-58 may attenuate the immune response of lymphocytes, potentially limiting a critical endogenous antitumor mechanism. However, immunostaining of CT26 cell-derived tumors revealed no substantial differences in CD8^+^ cells infiltration among treatment groups (Fig. 5A, Supplementary Fig. S2). Further, immunoexpression of PD-L1 was not markedly altered (Fig. 5A, Supplementary Fig. S3).

### Effects of KS-58 on kidney and liver

Finally, to further evaluate the safety of KS-58, we conducted histomorphological analyses of mouse kidney and liver (Fig. 5B). No obvious histological changes in any of the nephron segments were observed following treatment. In liver, hepatocellular hypertrophy with cytoplasmic eosinophilic change was observed following treatment with KS-58 alone and KS-58 plus anti-PD-1. However, there was no evidence of hepatocellular degeneration or necrosis in any of the treatment groups. Thus, hepatic hypertrophy appears to be within the range of adaptive reactions associated with drug metabolism rather than an adverse reaction.

## Discussion

The findings presented here and in our previous report^14^ show that the reversible K-Ras(G12D) inhibitor KS-58 can suppress the growth of tumors derived from PANC-1 pancreatic cancer cells and CT26 colorectal cancer cells expressing K-Ras(G12D) without serious side effects such as weight gain/loss, liver pathology, or kidney dysfunction. Further, these results provide proof of principle for the wider application of reversible K-Ras inhibitor to include cancers associated with other K-Ras mutations such as K-Ras(G12C), K-Ras(G12V).

A covalent inhibitor of K-Ras(G12C) recently granted regulatory approval for clinical use has demonstrated high target selectivity and shown to possess substantial therapeutic activity against lung cancer, especially when combined with an immune checkpoint inhibitor^6^. This K-Ras(G12C) inhibitor is reported to enhance tumor infiltration of effector T-cells and to downregulate PD-L1 expression^6^. Moreover, other Ras pathway inhibitors have shown additive anticancer efficacy when used in combination with immune checkpoint inhibitors^19–22^. Based on these reports, we expected that the anticancer efficacy of KS-58 would be enhanced by an immune checkpoint inhibitor, but unexpectedly, KS-58 plus anti-PD-1 was no more effective than KS-58 alone despite the confirmed inhibitory activity of KS-58 against K-Ras(G12D) function in vivo and the demonstrated non-interference of this effect by anti-PD-1. KS-58 was also relatively stable in liver microsomes and whole blood, which should enhance bioavailability of KS-58 in tumor and thus the potential for therapeutic interactions with anti-PD-1. However, a large fraction of KS-58 in whole blood eventually bound to the surface or accumulated within blood cells, which could have limited potential interactions with anti-PD-1. One possibility is that the additive or synergistic effects of KS-58 plus anti-PD-1 are mediated by antitumor immunity and that KS-58 entry into leukocytes disrupts this process, but immunostaining of CT26 tumors revealed that KS-58 did alter effector T-cell (CD8^+^) tumor infiltration or PD-L1 expression levels. Therefore, the reason for this lack of combined activity with anti-PD-1 remains unclear. It is still possible that KS-58 reduces the antitumoral lymphocyte response after infiltration, a potential mechanism requiring further study.

The t_1/2_ of KS-58 in mice was relatively short (~ 30 min) despite the relatively high degree of plasma protein binding (Fig. 4E), which would be expected to buffer any concentration decrease. However, the rate-limiting factor determining half-life could be elimination by metabolism within organs as plasma protein binding is likely non-selective and rapidly reversible. Another bicyclic peptide of similar molecular weight to KS-58 and also resistant to protease degradation demonstrated a similar t_1/2_ of about 30 min following intravenous injection^23^. To elongate t_1/2_, more selective plasma protein-binding (e.g. albumin) with slower off-rates are required^24^. Although the detailed metabolic pathway is not clear, histomorphological analysis suggested that KS-58 is metabolized in liver. While this relatively short t_1/2_ limits the exposure time to target tumor tissue, it also reduces exposure to healthy tissue, thereby decreasing the risks of potentially serious side effects. We previously reported that the cell membrane permeability of KS-58 may be enhanced in weakly acidic environments such as tumor tissues^14^, a property that could contribute to the selective anticancer activity and limited side effects profile observed in this study.

Combination treatments consisting of a K-Ras inhibitor plus an immune checkpoint inhibitor like anti-PD-1 or Ras pathway inhibitor (EGFR, MEK, SHP2, and SOS1 inhibitor) are expected to have additive or synergistic anticancer activities. Unfortunately, KS-58 did not show additive or synergistic anticancer activity in combination with anti-PD-1 in this animal study. On the other hand, KS-58 has demonstrated additive anticancer activity in combination with the therapeutic DNA synthesis inhibitor gemcitabine^14^. Further studies are required to determine if the anticancer efficacy of KS-58 is additive or synergistic with other Ras pathway inhibitors.

In summary, the K-Ras(G12D)-inhibitory bicyclic peptide KS-58 dose-dependently inhibited the proliferation of CT26 colorectal cancer cells expressing K-Ras(G12D) and suppressed the growth of CT26-cell-derived tumors in mice, consistent with previous reports that this compound can inhibit the proliferation of a pancreatic cancer cell line expressing K-Ras(G12D). Unfortunately, the combination of KS-58 and anti-PD-1 was no more effective than KS-58 alone. Further studies are required to assess possible additivity or synergy with other immune checkpoint inhibitors, chemotherapeutics, and Ras pathway inhibitors.

## Supplementary Information

Supplementary Figures.

## Data Availability

All data generated for this study are contained in the manuscript. Raw data are available from the corresponding author upon reasonable request.
