# Supplementary material for: The K-Ras(G12D)-inhibitory peptide KS-58 suppresses growth of murine CT26 colorectal cancer cell-derived tumors
Source: Sci Rep. 2022 May 17;12:8121. doi: 10.1038/s41598-022-12401-3 (PMC9114382; doi:10.1038/s41598-022-12401-3)
Supplement: Supplementary file 1 — Supplementary Figures. [file 41598_2022_12401_MOESM1_ESM.pdf]

The K-Ras(G12D)-inhibitory peptide KS-58 suppresses growth of murine CT26 colorectal cancer cell-derived tumors

Kotaro Sakamoto*[,1], Bangzhong Lin[2], Kazuto Nunomura[2], Takeshi Izawa[3], and Shinsaku Nakagawa[2,4]

[1]Research & Development Depertment, Ichimaru Pharcos Company Limited, 318-1 Asagi, Motosu, Gifu 501-0475, Japan

[2]Center for Supporting Drug Discovery and Life Science Research, Graduate School of Pharmaceutical Science, Osaka University, 1-6 Yamadaoka, Suita, Osaka 565-0871, Japan

[3]Laboratory of Veterinary Pathology, Osaka Prefecture University, 1-58 Rinku-Orai-Kita, Izumisano, Osaka 598-8531, Japan

[4]Laboratory of Biopharmaceutics, Osaka University, 1-6 Yamadaoka, Suita, Osaka 565-0871, Japan

*Corresponding author:

Kotaro Sakamoto

E-mail: sakamoto-kotaro@ichimaru.co.jp, weidlichk58@gmail.com

Supplementary Figure S1. Full length blots of Figure 3**.**

pERK

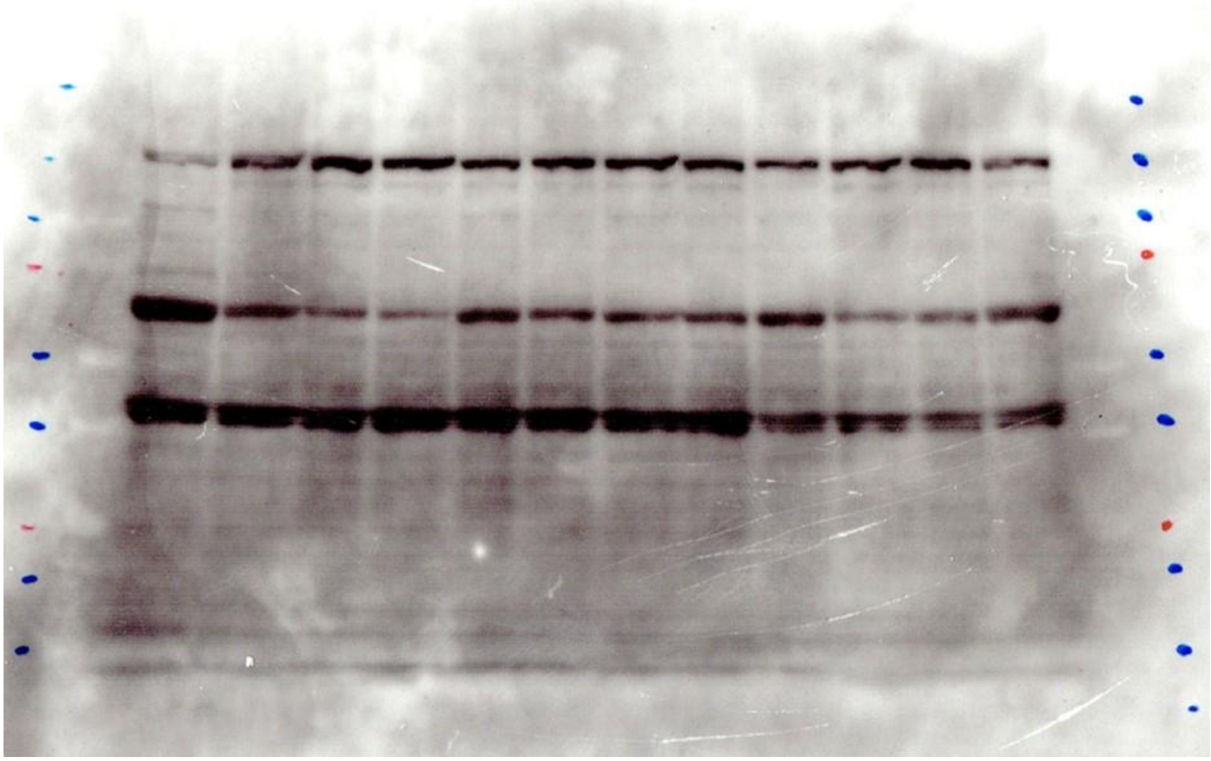

ERK

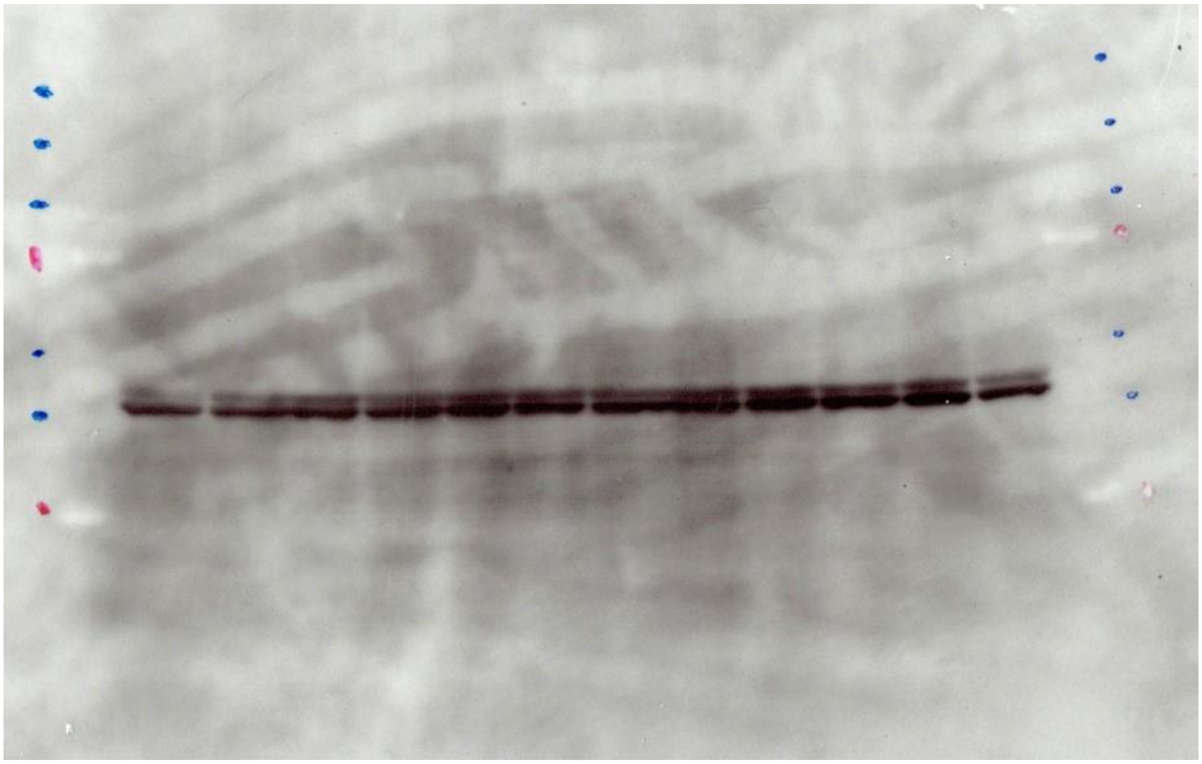

GAPDH

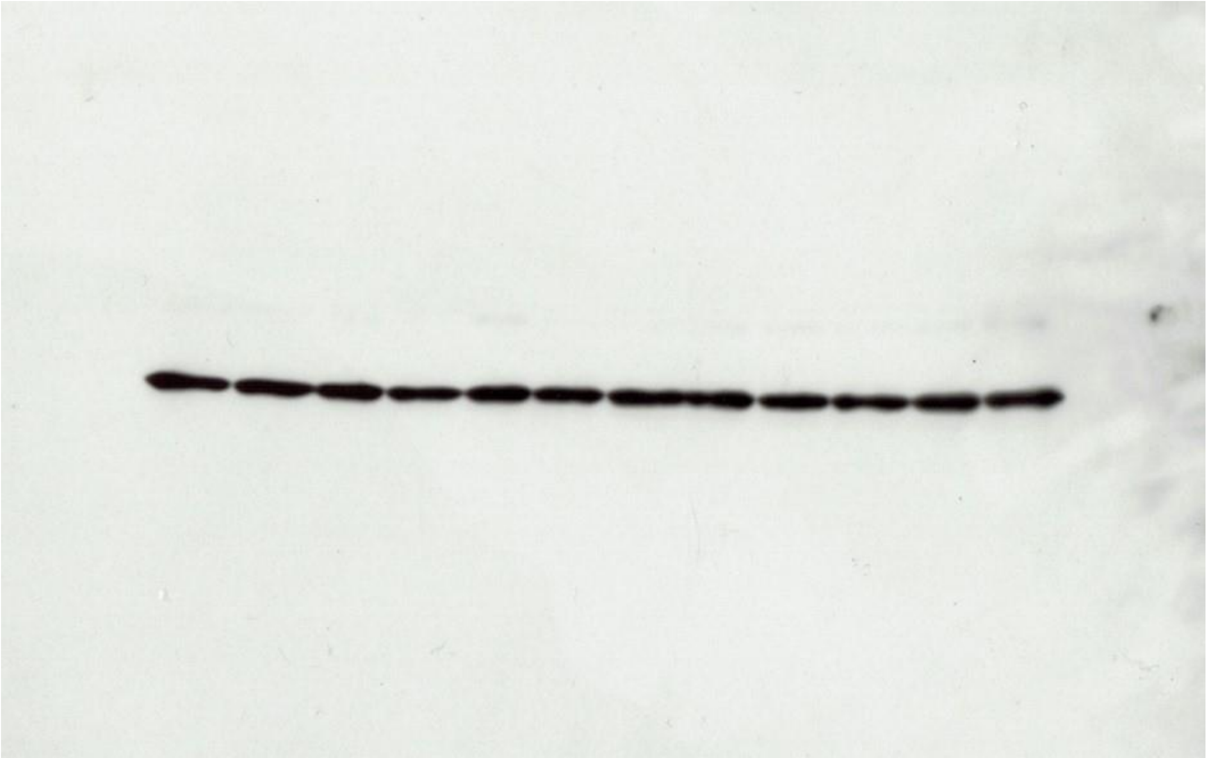

Supplementary Figure S2. CD8^{+} cells quantification of Figure 5A**.**

HE-stained slides were scanned with Olympus Virtual Slide VS-120, and CD8+ cells were counted in 5 fields of view at 20× objective lens (field of view area = 0.090798 mm^2). The measurement field of view was randomly selected from the area slightly medial to the limbus of the mass, excluding the necrotic nests in the center of the mass.

Group	Sample ID	Positive cells in the field of vision using objective lens (20-fold)					Average	Positive cell count/mm^2
		Field of vision 1	Field of vision 2	Field of vision 3	Field of vision 4	Field of vision 5		
Vehicle	SCT01	26	46	36	29	23	32.0	352.4
	SCT02	23	24	32	19	39	27.4	301.8
	SCT03	21	20	20	10	8	15.8	174.0
Anti-PD-1 Ab	SP01	30	15	29	23	17	22.8	251.1
	SP02	19	25	33	30	33	28.0	308.4
	SP03	48	35	43	27	43	39.2	431.7
KS-58 (high)	SH01	35	27	30	21	12	25.0	275.3
	SH02	38	34	25	16	17	26.0	286.3
	SH03	32	25	35	24	32	29.6	326.0
KS-58 (high) + anti-PD-1 Ab	SHP01	30	47	38	30	20	33.0	363.4
	SHP02	17	30	47	22	23	27.8	306.2
	SHP03	23	28	26	20	30	25.4	279.7

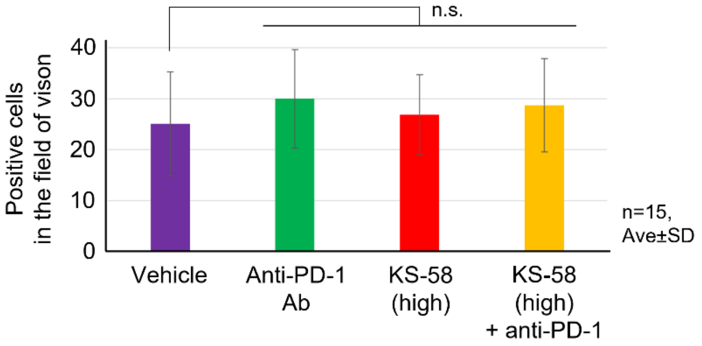

Supplementary Figure S3. PD-L1 expression levels in tumor cells of Figure 5A**.**

The entire specimen was observed under an optical microscope and scored for immunoreactivity. Necrotic areas were excluded from evaluation.

Group	Sample ID	Score
Vehicle	SCT01	3+
	SCT02	3+
	SCT03	3+
Anti-PD-1 Ab	SP01	1+
	SP02	2+
	SP03	2+
KS-58 (high)	SH01	1+
	SH02	1+
	SH03	1+
KS-58 (high) + anti-PD-1 Ab	SHP01	2+
	SHP02	1+
	SHP03	2+

Immunoreactivity score
0; absent
1+; slight
2+; mild
3+; moderate
4+; marked
